# Depressive Symptoms in Adults with Obesity and Their Associations with Sociodemographic, Health and Lifestyle Factors: A Secondary Analysis of a Cross-Sectional Study

**DOI:** 10.3390/jcm15207929

**Published:** 2026-10-14

**Authors:** Martyna Głuszek-Osuch, Elżbieta Cieśla, Kamila Sobaś, Dorota Kozieł, Stanisław Głuszek, Edyta Suliga

**Affiliations:** 1 Institute of Health Sciences, Collegium Medicum, Jan Kochanowski University, 25-369 Kielce, Polandelzbieta.ciesla@ujk.edu.pl (E.C.); kamila.sobas@ujk.edu.pl (K.S.);; 2 School of Public Health, Centre of Postgraduate Medical Education, 01-826 Warsaw, Poland; 3 Institute of Genetics and Animal Biotechnology, Polish Academy of Sciences, Jastrzębiec, 05-552 Magdalenka, Poland; 4 Department of Surgery, Hospital of the Ministry of Interior and Administration in Kielce, 25-375 Kielce, Poland; 5 Department of Surgical Oncology, Holy Cross Cancer Centre in Kielce, 25-734 Kielce, Poland

**Keywords:** depressive symptoms, degree of obesity, stressful life events, multimorbidity, lifestyle

## Abstract

**Background/Objectives**: Research indicates a bidirectional association between obesity and depression. The co-occurrence of both disorders has serious clinical implications. The aim of this study was to assess the prevalence of self-reported depressive symptoms (DS) in a group of men and women with obesity and to identify the health factors, sociodemographic factors and lifestyle elements associated with the severity of DS among these persons. **Methods**: The participants in the study were a group of 3742 persons aged 42–65 years with a BMI ≥ 30.0 kg/m^2^. A multivariate negative binomial regression analysis was performed in order to determine the expected number of DS. **Results**: Almost 28% of the participants with obesity showed at least four DS. The factors with the strongest associations with the number of DS were experiencing stressful events over the past year (IRR = 1.28, 95% CI: 1.23–1.33, *p* < 0.001), gender (IRR = 0.82, 95% CI: 0.79–0.85, *p* < 0.001) and multimorbidity (IRR = 1.10, 95% CI: 1.06–1.14, *p* < 0.001). The prevalence of DS was also associated with some socioeconomic factors (place of residence, education and marital status) and lifestyle factors (smoking, TV-viewing time, sleep duration and regular participation in organised groups or associations). **Conclusions**: Further longitudinal and intervention studies are necessary in order to better understand the factors related to the prevalence of DS in persons with obesity.

## 1. Introduction

Depression is a growing health and social problem. The number of patients with depression is estimated to have exceeded 332 million worldwide, including 5.7% of adults (4.6% of men and 6.9% of women) [1]. Depression is also considered to be one of the most commonly underdiagnosed conditions in clinical practice [2]. Many patients show a cluster of depressive symptoms (DS) that do not meet the diagnostic criteria for the disorder but may develop into full-blown depression. Although many studies on the risk factors of depression have been conducted, their results are ambiguous. Bidirectional associations between multimorbidity related to physical health and DS have been observed [3]. Most studies have also confirmed that depression is more prevalent among women than men [2,4,5,6] and that its prevalence increases with age [7]. On the other hand, some studies indicated a U-shaped relationship between age and depression [8]. Depressive symptoms were more prevalent in single persons than in persons living in a steady relationship [9,10,11]. Adults also showed associations between the number of their children and depression; however, these findings were inconsistent and different across countries [9,12,13]. Depressive symptoms are usually more prevalent in persons with low education [6,14,15] and a low economic status [5,11,16]. A recent analysis of longitudinal studies conducted across 20 countries showed that an unhealthy lifestyle accounted for 62.8% of the relationship between a low socioeconomic status and depression in adults aged ≥50 years [17]. A higher prevalence of DS was strongly associated with a lack of physical and social activity, as well as the consumption of alcohol, smoking, an unbalanced diet and insufficient sleep [17,18]. Much less is known about the factors preventing depression [19]. Some publications indicate that some dietary patterns [20,21] and components of the diet [22,23] may have an antidepressive effect. However, clinical studies on the use of supplementation and dietary interventions have yielded inconsistent results in this regard [21,24,25]. Adults who engaged in leisure-time physical activity displayed less severe DS compared to less active persons [26]. However, the significance of each behavioural factor may differ depending on gender, age and the country in which a study was conducted [17]. Most researchers agree that depression is much more prevalent in persons with excessive weight than in those with normal weight [27,28,29], and point out the existence of a bidirectional relationship between obesity and depression, emphasising their common psychological foundation and pathophysiological similarities [30,31,32,33]. Some researchers have suggested that obesity and depression may be comorbidities, i.e., that the two disorders often co-occur, but are not directly linked to each other and can be attributed to bidirectional relationships and common genetic factors [34]. The co-occurrence of obesity and depression has significant clinical implications: it impacts the patient’s health, self-esteem and social functioning, and may also make it difficult to treat either disorder on its own while increasing healthcare expenditures [35,36]. The research to date has focused primarily on comparing the prevalence of depression and its factors among persons with normal weight, overweight and obesity, but no studies have been conducted on the prevalence of DS in a population consisting exclusively of persons with obesity. The aim of this study was to estimate the prevalence of self-reported DS in a group of men and women with obesity and to identify the health, sociodemographic and lifestyle factors related to the burden of DS in persons with obesity. The study also aimed to determine whether gender moderates the relationship between these factors and DS.

## 2. Materials and Methods

### 2.1. Ethical Approval

The study received ethical approval from the Ethics Committee of the Cancer Centre and Institute of Oncology in Warsaw, Poland (Decision Number: 69/2009/1/2011—data collection). All the participants provided written informed consent to participate in the study. The study was also approved by the Bioethics Committee at the Faculty of Health Sciences, Jan Kochanowski University in Kielce, Poland (Decision Number: 45/2016—data analysis).

### 2.2. Study Design

A secondary analysis of the results of a cross-sectional study on the health of adult residents of Kielce and the Kielce Powiat was performed (the POlish-Norwegian Study project, PONS) [37]. Sociodemographic, health and lifestyle data were collected using direct interviews with structured questionnaires. The participants’ weights and heights were measured. The study was conducted between 2010 and 2012 in the medical institutions participating in the project by qualified and trained personnel.

### 2.3. Participants

**Figure 1:**
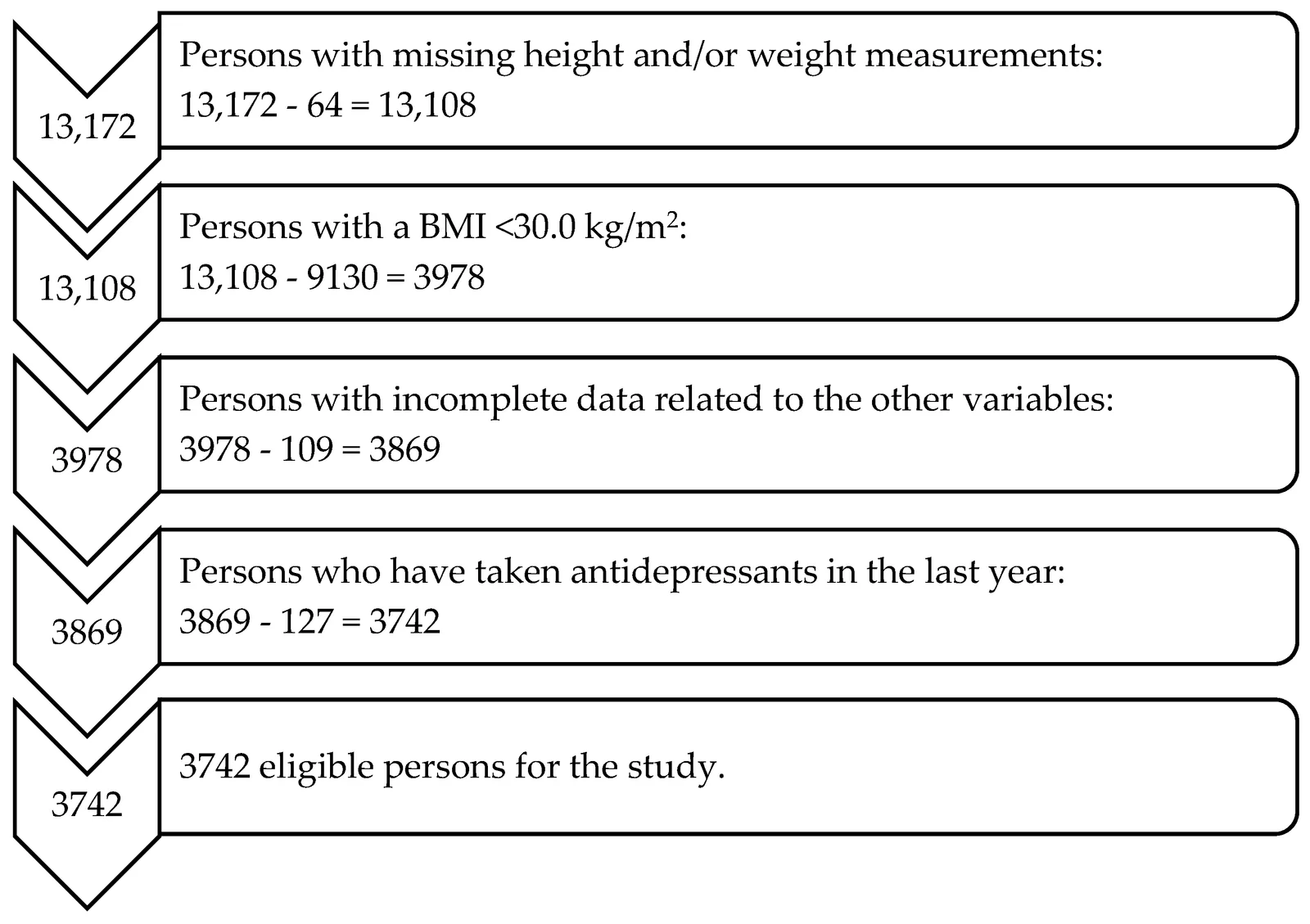
Flow chart for exclusion criteria.

Recruitment for the project was open and based on voluntary applications in response to public announcements originally directed at adults aged 45–64 years [37,38]. Ultimately, data from 13,172 persons aged between 37 and 66 years were collected. Sixty-four persons were excluded who did not undergo the height and/or weight measurements; subsequently, 9130 persons were also excluded with a BMI < 30.0 kg/m^2^, and there were 109 persons with incomplete data related to the other variables (Figure 1). In addition, 127 participants who had used antidepressants over the past year were excluded. The inclusion criteria were met by 3742 persons aged 42–65 years (1310 men).

### 2.4. Sociodemographic Data

Sociodemographic variables included gender (male, female), age (years), place of residence (the city of Kielce, 200,000 residents; village, the Kielce Powiat), education (elementary or vocational; high school; higher), number of children (0; 1; 2; ≥3) and marital status (married or in a steady relationship; single; widower/widow).

### 2.5. Health

Height and weight measurements were used to calculate the BMI (kg/m^2^) and to determine the experimental group (BMI ≥ 30.0 kg/m^2^) and the three degrees of obesity: Degree I (BMI = 30.0–34.9 kg/m^2^), Degree II (BMI = 35.0–39.9 kg/m^2^) and Degree III (BMI ≥ 40.0 kg/m^2^). Multimorbidity was defined as a diagnosis of at least two chronic diseases (diabetes, neoplastic disease/(cancer), coronary artery disease (ischaemic heart disease)/angina pectoris/myocardial infarction, circulatory failure, hypertension, stroke, asthma, chronic obstructive pulmonary disease or other), as declared by the participants (yes, no). The participants did not have a formal diagnosis of depression confirmed by a physician. Depressive symptoms were self-reported. The participants responded to eight questions concerning the occurrence of symptoms over the preceding 12 months (yes, no). The eight items used in the present study were adapted from the assessment of depressive symptoms used in the Prospective Urban and Rural Epidemiological (PURE) study, which was based on the Short-Form Composite International Diagnostic Interview (CIDI-SF) for major depressive disorders. The CIDI-SF is derived from the WHO Composite International Diagnostic Interview, and previous validation studies have supported its use for identifying depressive symptom burden [39,40]. Based on validation studies from the USA and Canada, a threshold of ≥4 DS was identified as predictive of major depressive disorder; therefore, this threshold was used in the PURE study. The reliability of the questionnaire in terms of its internal consistency was assessed based on Cronbach’s alpha, which amounted to 0.81 in the analysed population, indicating a good consistency of the responses to each item on the scale [38]. In our study, DS were assessed using eight dichotomous items (0 = symptom absent, 1 = symptom present). The responses were summed to obtain the number of self-reported depressive symptoms (range 0–8). The measure was used to quantify self-reported depressive symptom burden and was not interpreted as a diagnostic measure of clinical depression. The participants were also asked about the use of antidepressants over the last year (yes, no) and whether they had experienced any stressful life events in the last year, such as a serious disease or injury, violence, loss of employment, hunger, death or a serious disease of a close family member, or separation or divorce (yes, no).

### 2.6. Diet

A validated semiquantitative food frequency questionnaire designed for the PURE study was applied [41]. The participants reported how often they had consumed servings of specific food products over the last year by selecting one of the following response categories: 6 times a day or more, 4–5 times a day, 2–3 times a day, once a day, 5–6 times a week, 2–4 times a week, once a week, 1–3 times a month, less than once a month or never. Data on the consumption of fruit, vegetables, sugar, sweets and sweetened beverages were used in the analyses. The reported food frequencies and serving sizes were converted into average daily intakes. Each participant’s weekly consumption of pure ethanol was calculated based on its content in each type of alcoholic beverage. The consumption of alcohol was assessed according to three categories: non-drinkers (over the last year); small to moderate drinkers (0.1–30.0 g of ethanol/day for men and 0.1–15.0 g of ethanol/day for women); and large drinkers (>30.0 g of ethanol/day for men and >15.0 g of ethanol/day for women).

### 2.7. Lifestyle

The respondents were divided into nonsmokers, current smokers (every day) and past smokers (having not smoked for six months at the time of the study). Physical activity was assessed using the International Physical Activity Questionnaire. The respondents were asked if they satisfied the WHO recommendations for physical activity in adults (yes, no) [42]. The TV-viewing time was assessed based on the responses to the question: ‘How much time do you spend at home watching TV per week?’. The participants were divided into two groups: TV-viewing time <21 h/week and ≥21 h/week. The declared sleeping time was registered as full hours and analysed according to the following categories: ≤6 h/day, 7–8 h/day and ≥9 h/day. Furthermore, the participants declared their memberships in organised social or religious groups and associations and their regular attendance at them (at least once per month) (yes, no).

### 2.8. Statistical Analysis

Categorical variables are shown as numbers and percentages. Continuous variables are presented as the mean, standard deviation (*X* ± *SD*) and median (*Me*). DS-items were dichotomously coded (0 = symptom absent, 1 = symptom present) and summed to obtain the total number of self-reported depressive symptoms (range 0–8). The resulting variable was treated as a bounded count outcome. The distribution of the symptom count showed substantial overdispersion at the descriptive level (mean = 2.32, variance = 5.22). Therefore, negative binomial regression, which allows the conditional variance to exceed the conditional mean, was used to model the number of self-reported depressive symptoms. In order to determine the relationship between sociodemographic, health and lifestyle factors and the expected number of DS (the dependent variable), a multivariate negative binomial regression was used. Incidence rate ratios (IRRs) and 95% confidence intervals (CIs) were calculated. The Akaike information criterion (AIC) and the scaled Pearson’s coefficient of dispersion (scaled Pearson χ^2^/df) were also calculated as measures of the model fit. The Type III likelihood-ratio test (LR) was used to establish whether a given factor contributed significant information to the overall model after all other variables had been included. The independent qualitative variables were: place of residence (ref.: village), education (ref.: higher), marital status (ref.: married or in a steady relationship), number of children in the family (ref.: 0), multimorbidity (ref.: no), a stressful event in the past year (ref.: no), regular participation in organised groups or associations (ref.: yes), sleep duration (ref.: 7–8 h/day), smoking (ref.: nonsmokers), alcohol consumption (ref.: none), degree of obesity (ref.: Class I), meeting the WHO recommendations for physical activity (ref.: yes) and TV-viewing time (ref.: <21 h/week). The quantitative independent variables were the participants’ age and the consumption of fruits, vegetables and sweets. Interactions between gender and individual predictors were tested separately each time, by adding a single “gender × predictor” interaction term to a fully adjusted negative binomial regression model containing the same set of main effects. The significance of the interaction was assessed based on the LR test. Associations were assumed to be statistically significant at *p* < 0.05. Statistical analyses were performed using the STATISTICA 13.3 software package.

## 3. Results

### 3.1. General Characteristics of the Participants

The most numerous subgroups among the participants were women (64.99%), persons with Degree I obesity (72.80%), living in urban areas (56.28%), with secondary (39.93%) or primary education (38.38%), living in stable relationships (81.73%) and having two children (45.56%) (Table 1). Over 42% of the participants showed multimorbidity (at least two concurrent chronic diseases). Only 7.35% of the persons with obesity met the physical activity recommendations for adults, and 27.45% spent at least 21 h/week watching TV. The mean sleep duration was 7.16 ± 1.23 h/day (Me = 7.00). The mean consumption of fruit was 1.71 ± 1.06 servings/day (Me = 1.56), while the mean for vegetables was 2.81 ± 1.31 servings/day (Me = 2.63). The total consumption of sugar, sweets and sweetened beverages was 3.22 ± 3.10 servings/day (Me = 2.44). Over 80% of the participants declared consuming a small or moderate amount of alcohol. Current smokers made up 14.32% of the group, with former smokers making up 39.42%.

The most often declared DS was fatigue and a lack of energy to act, reported in almost half of all participants (Table 2).

### 3.2. Results of the Negative Binomial Regression Analysis

A negative binomial regression analysis showed that men had 18% fewer expected DS than women, after the inclusion of the other variables within the model (IRR = 0.82, 95% CI: 0.79–0.85, *p* < 0.001) (Table 3). Living in a city was also associated with fewer DS (by 9%) than living in a village. However, a higher than expected number of DS was found among persons with a primary school education (15% higher than those with a higher education), those living without a steady partner (15% higher), those with many children (7% higher), those with multiple chronic conditions (10% higher), as well as those who had experienced stressful events within the past year (28% higher) and those who did not regularly participate in organised groups or associations (8%). Furthermore, current smokers showed a higher prevalence of DS than nonsmokers (by 10%), as well as persons with a long TV-viewing time (by 5%) and a sleep duration shorter than seven hours/day (by 11%). Although a higher number of DS was also associated with a lower consumption of sweets, this result should be interpreted with caution due to its marginal significance. No significant differences were found in DS severity depending on age, degree of obesity, consumption of vegetables, fruits and alcohol, and adherence to the WHO recommendations on physical activity. The results of the LR test yielded the same statistical significance classification for 17 of the 18 predictors (Table S1). The only exception was the number of children, which did not contribute significant information to the model as a whole once all the other variables had been included. No interaction between gender and the other independent variables was found to be statistically significant (Table S2). The analyses conducted separately for each gender confirmed that men living in urban areas had fewer expected DS compared to those living in rural areas. A higher number of DS was associated with multimorbidity, current smoking and experiencing stressful events during the past year (Table S3). Women living in an urban area showed fewer expected DS compared to those living in a rural area, whereas a higher number of DS was observed among women with the lowest level of education, those without a steady marriage or relationship, those with multiple chronic conditions, those who had experienced stressful events within the past year, those who did not regularly participate in organised groups or associations and those who consumed more fruit (Table S4).

## 4. Discussion

The number of studies on the prevalence of DS in Poland is limited. To the best of our knowledge, this is the only study describing the prevalence of DS exclusively in individuals with obesity. The analyses presented in this article demonstrated that almost 28% of the study participants with obesity reported at least four DS. This percentage is similar to the prevalence in other populations, regardless of the definition of DS and the various tools employed to identify DS. A study conducted in Saudi Arabia found that 41.7% of participants with obesity aged 18 to 65 years had moderate to severe depression, as diagnosed using the Patient Health Questionnaire 9 (PHQ-9) [43]. In the 40–64 years age group from the US, depression/anxiety (identified based on the ICD-10-CM/ICD-10 diagnosis codes) occurred in 39% of individuals with obesity and 27.4% of individuals without obesity [44]. However, any comparisons between the prevalence of DS in the analysed group and their prevalence in other populations should be interpreted with caution, due to the different tools used in the studies. Significant gender-wise differences in the prevalence of DS were noted. The available literature has confirmed fairly unambiguously that DS are more prevalent in women than in men [2,5,6]. An analysis of gender-wise differences in DS among individuals aged 50+ years in Europe showed that women reported more DS than men across all regions, with the highest overall difference between genders occurring in Southern Europe [45]. Although the existence of a somatic subtype of depression in women that could explain the gender differences in depression was not confirmed, the authors’ review of the literature indicated that certain somatic DS were reported more frequently by women than by men, albeit not consistently across all studies [46]. It has been suggested that gender differences in the prevalence of DS may be related to anxiety, which is more common in women than in men, as well as a tendency to ruminate on problems more frequently, higher levels of stress and lower levels of fulfilment associated with traditional male or female gender roles. The results of a two-year-long observation indicated that the course of major depressive disorder worsened linearly with age, and the worst results related to the course of severity of the depression and remission time were observed among persons aged ≥70 years [7]. However, some studies have indicated the existence of a U-shaped relationship between age and the prevalence of DS, with a trend towards stabilisation at the age of 30–69 years [8]. The limited age range in the sample (42–65 years) may have caused the lack of observable associations between DS and age.

Persons living in a village declared DS more often than those living in a city. Other studies have also confirmed that city residents display better mental health indicators than village residents [47], which is most likely the result of the low socioeconomic status of village residents [16] and poorer prospects for finding well-paid employment in their place of residence, especially in the case of women, who are burdened with additional household and family responsibilities more often than men. Likewise, low education was related to much more frequent declarations of DS compared to persons with higher education. This relationship is also consistent with the results of earlier research conducted in Central and Eastern Europe [14]. Education is a well-established predictor of both physical and mental health. A good education may not only be associated with a higher economic status, but can also help to equip an individual with better knowledge, a sense of personal control and the ability to cope with stress. Persons without a steady relationship, especially women, presented more DS than those in stable relationships, which suggests that a life partner may be a source of emotional support for women in stressful situations, as well as a source of financial support, thereby reducing the risk of depression. This finding is consistent with the results of most studies that have been conducted to date [10,11]. The expected number of DS in persons with three or more children was higher compared to those without children. An international cross-sectional analysis performed in 20 countries suggested that the relationship between the number of children and depression differed depending on the region, taking on a U-shape in Europe [12]. Having more dependent children can lead to greater financial hardship for families and exacerbate social exclusion. However, this finding should be approached with caution, because this factor did not contribute significant information to the model after all the other variables had been included.

In the present study, stressful events experienced over the last year were found to show the strongest association with the presence of DS in both men and women with obesity. Studies have also frequently established that experiencing stressful events is one of the main factors in releasing subthreshold DS and a major depressive disorder [48,49], and that women reported more negative life events than men [50]. Despite many earlier findings indicating that women may be more susceptible to stressors and have less effective strategies of coping with them [51], the results of the more recent investigations have been ambiguous. Anderson et al. showed that stressful events were associated with DS equally in men and women [48]. Conversely, Shi et al. observed that women who had experienced multiple stressful events showed a stronger association with DS than men, whereas the relationship with major depressive disorder was stronger in men than in women [49]. However, when comparing the present study and studies conducted by other authors, it should be borne in mind that there are cultural differences in experiencing stressors and the methods of coping with stress. Another factor strongly associated with DS in the studied group was multimorbidity. The co-occurrence of DS and other chronic diseases is well-documented in the literature [52,53,54]. However, while most studies on the subject have focused on elderly persons, similar associations also occur at a younger age, especially in individuals with physical disorders involving intense pain [55].

Low-intensity chronic inflammation is believed to be a biological link between obesity and depression. Adipose tissue, acting as an endocrine organ, releases pro-inflammatory cytokines, such as interleukin-6 (IL-6) or tumour necrosis factor (TNF-α), and persons with obesity have elevated levels of inflammatory markers [56]. These molecules cross the blood–brain barrier, causing inflammation to occur in the central nervous system (neuroinflammation), which may affect brain function by means of neuroimmunological interactions, disrupted neurotransmitter metabolism, activation of the hypothalamic–pituitary–adrenal (HPA) axis and impaired neuroplasticity, leading to the onset of DS [57,58]. The developmental mechanisms of DS as the effect of chronic inflammation differ between genders. In men, chronic inflammation is more frequently related to visceral obesity and metabolic disorders such as insulin resistance, which have a strong activating effect on the HPA axis. Conversely, in women, inflammatory activity more often co-occurs with hormonal and immunological factors, which have a stronger modifying effect on the response of the central nervous system to stress [59,60]. Moreover, obesity is also a factor in the risk of multimorbidity [61]. The co-occurrence of multiple chronic diseases exacerbates systemic oxidative stress and inflammation, while biological inflammatory pathways serve as a link between physical multimorbidity, deteriorating mental health and an increased likelihood of DS [30,62]. The associations between obesity, multimorbidity and DS also have a genetic foundation [63,64]. Research has demonstrated a relationship between the polymorphisms of the genes encoding some pro-inflammatory cytokines (IL-1β) and the manifestation of specific DS, which confirms the mechanisms that link chronic inflammation with DS severity [65]. The associations between the degree of obesity and the severity of DS in the fully corrected model were not statistically significant. However, researchers predominantly believe that in cases of obesity, the risk of depression increases with an increase in BMI. In a prospective study conducted in Norway, the odds ratio for depression (measured using the Hospital Anxiety and Depression Rating Scale, HADS) increased linearly by 11% per standard deviation increase in BMI [66]. Likewise, in a study conducted in the US, a higher BMI, particularly in the case of Degree III obesity, was associated with an increased risk of DS (measured by the PHQ-9), with a stronger association observed in men than in women [57]. Tyrer et al. conducted a retrospective study in which they noted a dose–response relationship between an increase in BMI and depression; however, this relationship weakened at a BMI ≥ 50 kg/m^2^ [28]. Conversely, Luo et al. observed that Degree II obesity did not increase the severity of DS [67]. Consequently, the prevalence of DS does not always increase directly proportionally to an increase in BMI. It may also result from the fact that with age, the BMI becomes less reflective of the body fat percentage or from different personal resistance and/or potential adaptive mechanisms. The lack of a relationship between the degree of obesity and DS observed in the present study may have also resulted from the relatively small number of participants with higher degrees of obesity.

The results of this study showed that compared to nonsmokers, current smokers (especially men) have a higher risk of DS, which is consistent with the findings of other authors [68,69]. This relationship may be the effect of many different mechanisms. Research has indicated that smoking may contribute to the pathogenesis of DS by inducing chronic inflammation, oxidative stress and metabolic homeostasis disorders [70,71]. Furthermore, smoking may exacerbate mood disorders by disrupting the functions of key brain neurotransmitters, including 5-hydroxytryptamine and dopamine [72]. On the other hand, persons with DS may smoke as a coping mechanism for psychological stress.

Although a higher number of DS was also associated with a lower consumption of sweets, this finding should be interpreted with caution due to its marginal significance. A study conducted with a representative sample of adults in the US found that the overall consumption of sugar showed a positive correlation with a higher prevalence of DS in persons with obesity, but no such relationship was observed in persons with normal weight or who were overweight [73]. Although foods and drinks containing sugar are among the main factors contributing to gaining weight, they may also improve one’s mood and alleviate negative emotional states [74]. In the short term, eating sweets triggers a surge of dopamine and serotonin, thereby temporarily reducing anxiety and improving mood. The cross-sectional study could therefore capture such a relationship. More frequent consumption of sweets might also have been correlated with another factor that protects against depression (e.g., more frequent dining during meetings of organised groups or associations or higher income). The lower consumption of sweets in persons with a higher number of DS noted in this study may have resulted from the participants’ attempts to reduce their intake in order to lose weight. It should also be taken into account that individuals with obesity may have underreported the frequency of consuming sweet snacks due to feelings of guilt. The weak relationship between a lower consumption of fruit and a higher DS severity observed in women is consistent with the results of analyses indicating that a high consumption of some specific subgroups of fruit is generally considered to provide a protective effect against DS [22]. Although consuming alcohol may temporarily alleviate tension, it also negatively affects dopamine and serotonin concentrations, causes sleep disorders and increases the susceptibility to anxiety, thereby intensifying DS. However, this study did not confirm a relationship between the consumption of alcohol and the prevalence of DS, as noted by other authors [75], which may have resulted from a relatively small number of participants declaring a high consumption of alcohol. A study conducted in the U.S. showed that adults who engaged in ≥150 min per week of moderate-to-vigorous physical activity during their leisure time experienced lower levels of depression compared to less active individuals [26]. Another study found that higher activity levels were associated with fewer DS in overweight and/or obese individuals, but not in persons with a normal weight, suggesting that the BMI may moderate the relationship between physical activity and depression [76]. Certain symptoms of depression, including a lack of motivation, low mood and low energy, lead to reduced physical activity and weight gain, and consequently, obesity, creating a cycle in which obesity and depression reinforce each other. The associations between the adherence to the WHO recommendations for physical activity and the expected number of DS were not significant among the participants of this study. The only finding was that a longer TV-viewing time—that is, time spent sitting, reclining or lying down—was associated with a higher number of DS. These results suggest that physical activity and sedentary behaviour are two independent factors linked to our mental health in different ways. It can therefore be concluded that simply meeting WHO guidelines on physical activity does not negate the adverse effects of prolonged sitting. A short sleep duration (<7 h/day) was also markedly associated with a higher severity of DS compared to an optimal sleep duration, which is consistent with other reports [77]. Sleep deprivation may act as a neurobiological stressor by overactivating the stress response system and negatively affecting the areas of the brain involved in regulating emotions, mood and cognitive functions, thereby contributing to a susceptibility to depression [78]. Participation in organised social or religious groups was associated with a lower expected number of DS, which is consistent with the results of many other studies [79]. Moreover, many publications have confirmed that women use support groups and are open to seeking help more often and more willingly than men [80,81].

### Limitations and Strengths

One of the limitations of this study was an incomplete representation of the population. The participants were exclusively volunteers who lived in a specific geographic region, with women making up nearly 2/3 of the group. This sample composition may limit the generalisability of the results to the entire population and to other ethnic groups. Because of the cross-sectional design, it was also impossible to draw causal conclusions. Furthermore, the study lacked data on some factors that may potentially affect the prevalence of DS, such as weight change, chronic stressors, including weight-related stigma, low household income and poor living conditions [82]. The relatively long time interval between the data collection and publication may also affect the accuracy of certain findings concerning lifestyle and mental health, and limit the generalisability of the results to the current population. Moreover, the research tool employed in the study did not allow for a clinical diagnosis of depression. However, the questionnaire used in this paper has been successfully applied for the assessment of DS in other international studies [39,40]. The strengths of the study included a large number of persons with obesity determined based on measurements of their height and weight, rather than declared values. Furthermore, the group was ethnically uniform, which meant that the BMI ranges reflected a similar body fat percentage. The analyses were conducted separately for men and women, and took into account many of the potential risk factors for DS.

## 5. Conclusions

An analysis conducted among adults with obesity indicated that the factors that were most strongly associated with DS were: stressful events over the last year, gender and multimorbidity. The prevalence of DS was higher in women than in men, whereas the directions of the relationships observed between the analysed factors were similar in both genders. The degree of obesity did not differentiate the likelihood of DS. A higher number of DS was associated with some socioeconomic factors (living in a rural area, poor education and lack of a steady relationship) and lifestyle factors (current smoking, long TV-viewing time, short sleep duration, and no regular participation in organised groups or associations). However, further longitudinal and intervention studies are required in order to better understand the factors related to the prevalence of DS in persons with obesity and the mechanisms of their action.

## Figures and Tables

**Table 1:** Characteristics of the study participants.

Factors	Categories	*n*	%
Gender	Men	1310	35.01
	Women	2432	64.99
Age [years]	X ± SD (Me)	56.69 ± 5.09 (57.00)	
Place of residence	City	2106	56.28
	Village	1636	43.72
Education	Primary	1436	38.38
	Secondary	1494	39.93
	Higher	812	21.70
Marital status	In a steady relationship	3041	81.27
	Not in a steady relationship	701	18.73
Number of children	0	235	6.28
	1	460	12.29
	2	1705	45.56
	≥3	1342	35.86
Degree of obesity	I	2724	72.80
	II	789	21.08
	III	229	6.12
Multimorbidity	No	2160	57.72
	Yes	1582	42.28
Number of declared depressive symptoms (DS)	0	1115	29.80
	1	597	15.95
	2	562	15.02
	3	436	11.65
	4	306	8.18
	5	276	7.38
	6	200	5.34
	7	136	3.63
	8	114	3.05
Stressful life events	No	2869	76.67
	Yes	873	23.33
Consumption of fruit [servings/day]	X ± SD (Me)	1.71 ± 1.06 (Me = 1.56)	
Consumption of vegetables [servings/day]	X ± SD (Me)	2.81 ± 1.31 (Me = 2.63)	
Consumption of sweets [servings/day]	X ± SD (Me)	3.22 ± 3.10 (Me = 2.44)	
Consumption of alcohol [g ethanol/day]	None	628	16.78
	Small and moderate	3031	81.00
	Large	83	2.22
Smoking status	Nonsmokers	1731	46.26
	Current smokers	536	14.32
	Former smokers	1475	39.42
Meeting WHO recommendations for physical activity	Yes	275	7.35
	No	3467	92.65
TV-viewing time [h/day]	<21	2715	72.55
	≥21	1027	27.45
Sleep duration [h/day]	<7	1004	26.83
	7–8	2615	69.88
	≥9	123	3.29
Regular participation in organised groups or associations	Yes	240	6.41
	No	3650	93.59

**Table 2:** Descriptive statistics of the DS declared by the participants with obesity.

Have You Experienced the Following DS Over the Last 12 Months?	*n*	%
Were you sad, distressed or depressed?	818	21.83
Did you lose interest in things you used to enjoy (such as hobbies, work or other activities)?	802	21.40
Did you feel tired or lacked the energy to act?	1791	47.80
Did you gain or lose weight?	1026	27.38
Did you have more trouble sleeping than usual?	1362	36.35
Did you have more trouble concentrating/focusing than usual?	1124	30.00
Did you have thoughts about death (yours, a family member’s, general)?	968	25.83
Did you feel helpless or worthless?	806	21.51

**Table 3:** Factors related to the prevalence of DS in persons with obesity: results of a multivariate negative binomial regression analysis.

Variables	Categories	IRR	95% CI		*p*
			Lower	Upper	
Gender	Women—ref.				
	Men	0.82	0.79	0.85	<0.001
Age		1.00	0.99	1.00	0.601
Place of residence	Village—ref.				
	City	0.91	0.88	0.95	<0.001
Education	Higher—ref.				
	Secondary	0.98	0.93	1.02	0.332
	Primary	1.15	1.09	1.21	<0.001
Marital status	In a steady relationship—ref.				
	Not in a steady relationship	1.10	1.05	1.14	<0.001
Number of children	0—ref.				
	1	1.01	0.93	1.09	0.851
	2	0.98	0.93	1.04	0.569
	≥3	1.07	1.01	1.14	0.032
Degree of obesity	I—ref.				
	II	0.98	0.92	1.04	0.510
	III	1.05	0.96	1.14	0.322
Multimorbidity	No—ref.				
	Yes	1.10	1.06	1.14	<0.001
Stressful life events	No—ref.				
	Yes	1.28	1.23	1.33	<0.001
Consumption of fruit		0.97	0.94	1.01	0.115
Consumption of vegetables		1.01	0.99	1.04	0.281
Consumption of sweets		0.99	0.98	1.00	0.046
Consumption of alcohol	None—ref.				
	Small and moderate	1.05	0.96	1.14	0.286
	Large	0.97	0.83	1.14	0.708
Smoking status	Nonsmokers—ref.				
	Current smokers	1.10	1.04	1.17	0.002
	Former smokers	0.98	0.94	1.03	0.493
Meeting WHO recommendations for physical activity	Yes—ref.				
	No	1.03	0.96	1.09	0.433
TV-viewing time [h/day]	<21—ref.				
	≥21	1.05	1.01	1.09	0.010
Sleep duration [h/day]	7–8—ref.				
	<7	1.11	1.03	1.19	0.006
	>8	0.97	0.86	1.09	0.591
Regular participation in organised groups or associations	Yes—ref.				
	No	1.08	1.01	1.16	0.030

Model fit: AIC = 14,708.58; Pearson χ^2^/df = 0.940.

## Data Availability

The datasets used and/or analysed during the current study are available from the corresponding author upon reasonable request.

## Supplementary Materials

The following supporting information can be downloaded at: https://www.mdpi.com/article/10.3390/jcm15207929/s1. Table S1: Results of the Type III likelihood-ratio test. Table S2: Interaction of gender with other variables. Table S3: Factors associated with DS in men. Results of multivariable negative binomial regression. Table S4: Factors associated with DS in women. Results of multivariable negative binomial regression.

## Abbreviations

The following abbreviations are used in this manuscript:

DS	Depressive symptoms
BMI	Body Mass Index
IRR	Incidence rate ratio
CI	Confidence interval
PONS	POlish-Norwegian Study
PURE	Prospective Urban and Rural Epidemiological study
X ± SD	Arithmetic mean ± Standard Deviation
Me	Median
AIC	Akaike Information Criterion
LR	Type III likelihood-ratio test
WHO	World Health Organisation
PHQ-9	Patient Health Questionnaire 9
ICD-10-CM	International Classification of Diseases, Tenth Revision, Clinical Modification
HPA	Hypothalamic–pituitary–adrenal axis
