# Supplementary material for: Depressive Symptoms in Adults with Obesity and Their Associations with Sociodemographic, Health and Lifestyle Factors: A Secondary Analysis of a Cross-Sectional Study
Source: J Clin Med. 2026 Oct 14;15(20):7929. doi: 10.3390/jcm15207929 (PMC13706354; doi:10.3390/jcm15207929)
Supplement: Supplementary file 1 [file jcm-15-07929-s001.zip › jcm-4596084-supplementary.pdf]

Table S1. Results of the Type III likelihood-ratio test.

Factors	df	χ^2 LR	*p*
Gender	1	**103.280**	**<0.001**
Age	1	0.268	0.604
Place of residence	1	**25.230**	**<0.001**
Education	2	**30.945**	**<0.001**
Marital status	1	**17.967**	**<0.001**
Number of children	3	6.099	0.107
Degree of obesity	2	1.016	0.602
Multimorbidity	1	**29.954**	**<0.001**
Stressful life events	1	**173.929**	**<0.001**
Consumption of fruit	1	2.402	0.121
Consumption of vegetables	1	1.049	0.306
Consumption of sweets	1	**3.905**	**0.048**
Consumption of alcohol	2	2.278	0.320
Smoking status	2	**12.982**	**0.002**
Meeting WHO recommendations for physical activity	1	0.614	0.433
TV-viewing time	1	**6.589**	**0.010**
Sleep duration	2	**21.505**	**<0.001**
Regular participation in organised groups or associations	1	**4.695**	**0.030**

df – degrees of freedom; LR - Type III likelihood-ratio test

Table S2. Interaction of gender with other variables.

Interaction	df	LR_{χ^2}	p
Gender x Age	1	1.423	0.233
Gender x Place of residence	1	0.190	0.663
Gender x Education	2	3.080	0.214
Gender x Marital status	1	0.058	0.810
Gender x Number of children	3	3.485	0.323
Gender x Degree of obesity	2	0.515	0.773
Gender x Multimorbidity	1	3.269	0.071
Gender x Stressful life events	1	0.106	0.744
Gender x Consumption of fruit	1	0.576	0.448
Gender x Consumption of vegetables	1	0.269	0.604
Gender x Consumption of sweets	1	1.049	0.306
Gender x Consumption of alcohol	2	3.481	0.175
Gender x Smoking status	2	0.731	0.694
Gender x Meeting WHO recommendations for physical activity	1	0.135	0.713
Gender x TV-viewing time	1	0.081	0.775
Gender x Sleep duration	2	4.582	0.101
Gender x Regular participation in organised groups or associations	1	0.392	0.531

df – degrees of freedom; LR - Type III likelihood-ratio test

Table S3. Factors associated with DS in the men. Results of multivariable negative binomial regression.

Variables	Categories	IRR	95% CI		p
			lower	upper	
Age		0.99	0.98	1.00	0.118
Place of residence	Village - ref.				
	City	**0.90**	**0.84**	**0.97**	**0.005**
Education	Higher - ref.				
	Secondary	0.95	0.87	1.05	0.315
	Primary	1.09	0.99	1.20	0.078
Marital status	In a steady relationship – ref.				
	Not in a steady relationship	1.09	0.96	1.23	0.186
Number of children	0. - ref.				
	1	0.96	0.82	1.12	0.603
	2	0.92	0.82	1.03	0.145
	≥3	1.09	0.97	1.24	0.159
Degree of obesity	I - ref.				
	II	0.95	0.81	1.13	0.581
	III	1.08	0.82	1.41	0.590
Multimorbidity	No - ref.				
	Yes	**1.16**	**1.08**	**1.25**	**<0.001**
Stressful life events	No - ref.				
	Yes	**1.29**	**1.20**	**1.40**	**<0.001**
Consumption of fruit		1.00	0.93	1.07	0.975
Consumption of vegetables		1.00	0.95	1.05	0.952
Consumption of sweets		0.99	0.97	1.01	0.256
Consumption of alcohol	None – ref.				
	Small and moderate	1.04	0.91	1.18	0.592
	Large	0.88	0.71	1.09	0.237
Smoking status	Nonsmokers – ref.				
	Current smokers	0.96	0.88	1.05	0.385
	Former smokers	**1.16**	**1.03**	**1.30**	**0.011**
Meeting WHO recommendations for physical activity	Yes – ref.				
	No	1.05	0.92	1.20	0.444
TV-viewing time [h/day]	<21 – ref.				
	≥21	1.06	0.98	1.13	0.135
Sleep duration [h/day]	7-8 – ref.				
	<7	1.05	0.89	1.24	0.554
	>8	0.95	0.72	1.26	0.741
Regular participation in organised groups or associations	Yes - ref.				
	No	1.06	0.92	1.21	0.444

IRR - Incidence rate ratio

Table S4. Factors associated with DS in the women. Results of multivariable negative binomial regression.

Variables	Categories	IRR	95% CI		p
			lower	upper	
Age		1.00	0.99	1.01	0.649
Place of residence	Village - ref.				
	City	**0.91**	**0.88**	**0.95**	**<0.001**
Education	Higher - ref.				
	Secondary	0.99	0.94	1.04	0.606
	Primary	**1.18**	**1.11**	**1.25**	**<0.001**
Marital status	In a steady relationship – ref.				
	Not in a steady relationship	**1.10**	**1.05**	**1.14**	**<0.001**
Number of children	0. - ref.				
	1	1.03	0.94	1.13	0.504
	2	1.01	0.94	1.08	0.848
	≥3	1.05	0.98	1.13	0.148
Degree of obesity	I - ref.				
	II	0.99	0.93	1.06	0.828
	III	1.03	0.94	1.13	0.492
Multimorbidity	No - ref.				
	Yes	**1.07**	**1.03**	**1.11**	**<0.001**
Stressful life events	No - ref.				
	Yes	**1.27**	**1.22**	**1.33**	**<0.001**
Consumption of fruit		**0.96**	**0.92**	**0.99**	**0.038**
Consumption of vegetables		1.02	0.99	1.06	0.111
Consumption of sweets		0.99	0.98	1.00	0.167
Consumption of alcohol	None – ref.				
	Small and moderate	0.98	0.84	1.14	0.784
	Large	1.15	0.87	1.54	0.331
Smoking status	Nonsmokers – ref.				
	Current smokers	1.00	0.94	1.06	0.923
	Former smokers	1.07	1.00	1.16	0.066
Meeting WHO recommendations for physical activity	Yes – ref.				
	No	1.01	0.94	1.09	0.717
TV-viewing time [h/day]	<21 – ref.				
	≥21	**1.05**	**1.01**	**1.10**	**0.023**
Sleep duration [h/day]	7-8 – ref.				
	<7	**1.13**	**1.05**	**1.23**	**0.002**
	>8	0.97	0.85	1.11	0.693
Regular participation in organised groups or associations	Yes - ref.				
	No	**1.09**	**1.01**	**1.18**	**0.028**

IRR - Incidence rate ratio
